# HRV-Based Surrogate Classification in Surgical Environments Using Personalised Labelling and Sequence Modelling

**DOI:** 10.3390/s26154822

**Published:** 2026-07-30

**Authors:** Abdulaziz Almuhini, Viji Ahanathapillai, Zeeshan Raza, Imran Aslam, Abhilasha Patel, Charles Evans, Leandro Pecchia, Davide Piaggio

**Affiliations:** 1 Department of Biomedical Technology, College of Applied Medical Sciences in Al-Kharj, Prince Sattam bin Abdulaziz University, Al-Kharj 11942, Saudi Arabia; 2 Applied Biomedical Signal Processing and Intelligent eHealth (ABSPIE) Lab, School of Engineering, University of Warwick, Coventry CV4 7AL, UK; viji.ahanathapillai@warwick.ac.uk (V.A.); zeeshan-raza.raza@warwick.ac.uk (Z.R.); leandro.pecchia@unicampus.it (L.P.); davide.piaggio@warwick.ac.uk (D.P.); 3 Department of Colorectal Surgery, University Hospital Coventry and Warwickshire, Coventry CV2 2DX, UK; imran.aslam@uhcw.nhs.uk (I.A.); abhilasha.patel@uhcw.nhs.uk (A.P.); charles.evans@uhcw.nhs.uk (C.E.); 4 School of Engineering, Università Campus Bio-Medico di Roma, Via Álvaro del Portillo, 00128 Rome, Italy

**Keywords:** HRV, stress detection, surgical performance, wearable sensing, deep learning, personalised modelling, LOSO validation

## Abstract

Accurate monitoring of stress and workload in surgical environments is essential for improving performance and patient safety. Physiological signals, particularly heart rate variability (HRV), offer a promising approach for objective and continuous stress assessment. However, existing studies often rely on population-level models, limited temporal analysis, and evaluation strategies that may not reflect real-world generalisation. This study proposes an end-to-end pipeline for HRV-based classification of HRV-derived surrogate stress-like states in surgical settings, combining personalised feature extraction, surrogate label generation, and sequence-based deep learning. HRV data were collected from multiple surgeons during real colorectal procedures using wearable sensors and processed into minute-level feature representations. Stress labels were generated using subject-specific thresholds to account for individual physiological differences. Sequence-based models, including convolutional neural networks (CNNs) and long short-term memory (LSTM) networks, were evaluated across multiple temporal window lengths and validation strategies. The results show that mean-based Standard Deviation of Normal-to-Normal intervals (SDNN) provided the most consistent separation of physiological states. The highest-performing models were most frequently associated with temporal windows of 10–20 min, while CNN and LSTM architectures achieved comparable performance. Notably, in this dataset, leave-one-subject-out (LOSO) validation produced performance that was comparable to, and in some configurations higher than, pooled random splits, highlighting the influence of evaluation strategy and the potential benefits of personalised labelling. Overall, this work suggests that personalised HRV-based modelling and temporal sequence analysis may provide a feasible approach for identifying HRV-derived stress-like physiological states in real surgical environments. The findings also emphasise the importance of rigorous evaluation design when developing physiological machine learning systems.

## 1. Introduction

Modern surgical practice involves complex, high-stakes tasks that require constant attention, precise motor control, and rapid decision-making under time pressure. In this setting, stress and cognitive workload are not just background factors. Rather, they can directly influence clinical performance. Previous work has shown that acute stress can negatively affect both technical skills, such as accuracy and efficiency, and non-technical skills, including communication and coordination, which are essential for patient safety [1,2,3]. For this reason, there is growing interest in developing objective ways to monitor stress and workload in surgical environments, with potential applications in performance assessment, training, and intraoperative support.

Measuring stress reliably in real time remains challenging. Self-report tools are commonly used, but they are retrospective and cannot capture rapid changes during a procedure. Physiological signals provide a more continuous and objective alternative [4]. Among these, HRV is widely used as an indicator of autonomic nervous system activity, reflecting the balance between sympathetic and parasympathetic responses [5,6]. HRV is particularly appealing because it can be measured non-invasively and integrated into real-world monitoring systems. Recent developments in wearable sensing have made continuous physiological monitoring more practical outside controlled laboratory settings [7,8]. As a result, there has been increasing interest in short-term and ultra-short HRV analysis, which allows for higher temporal resolution and better alignment with real-world applications [9]. At the same time, these approaches come with limitations, as short-window HRV features are not always directly comparable to standard short-term measurements and require careful interpretation [6].

Despite these advances, several limitations still constrain the use of HRV for stress classification in surgical contexts. First, there is limited standardisation in how HRV is collected, processed, and interpreted, which makes it difficult to compare findings across studies [10]. Second, HRV varies substantially between individuals, yet many existing approaches rely on population-level models that do not account for subject-specific baselines. Third, most prior work focuses on aggregated or static features, with relatively little attention given to fine-grained temporal patterns, such as minute-level changes, using models that explicitly capture sequential dynamics. Finally, evaluation practices remain a concern. In particular, the use of random pooled data splits can lead to overly optimistic performance estimates, as data from the same individual may be present in both the training and testing sets, resulting in subject-level data leakage. In contrast, subject-wise validation provides a more realistic measure of generalisation [11,12].

To address these challenges, this study proposes an end-to-end pipeline for HRV-based classification of HRV-derived surrogate stress-like physiological states detection in surgical settings. The pipeline includes preprocessing of physiological signals, extraction of minute-level SDNN, and the generation of subject-specific surrogate stress labels based on individualised thresholds. While these labels do not represent externally validated ground truth, they provide a practical way to model stress-related physiological patterns in datasets where continuous expert annotation is not available.

Building on this pipeline, we examine the role of temporal dynamics in HRV-derived surrogate stress-like physiological state classification by evaluating sequence-based deep learning models, including CNNs and LSTM architectures, across multiple temporal window lengths. In addition, we compare different evaluation strategies, including pooled random splits and LOSO validation, to better understand how validation design affects model performance and generalisation. Because continuous psychological stress measurements were not available during surgery, the proposed framework employs HRV-derived surrogate stress-like physiological states as supervisory labels rather than independently validated measures of psychological stress.

The main contributions of this work are as follows: we present an end-to-end HRV processing and modelling pipeline tailored to stress and workload classification in surgical data. We introduce a personalised threshold-based approach for generating surrogate stress labels from physiological signals. We provide a systematic comparison of sequence-based models across multiple temporal resolutions, highlighting the role of temporal dynamics in the classification of HRV-derived surrogate stress-like physiological states. Finally, we establish a rigorous evaluation framework that demonstrates the impact of validation strategy on performance estimation and generalisation. Overall, this study aims to strengthen the methodological foundations of HRV-based surrogate stress-like physiological state classification in clinical environments. By combining personalised modelling, temporal analysis, and more rigorous evaluation strategies, it contributes toward the development of more reliable and practically applicable physiological monitoring systems for surgical performance assessment.

Building on existing research, HRV is commonly used as a non-invasive indicator of autonomic nervous system activity and stress, where lower HRV is generally linked to higher physiological stress levels [5,6,13,14]. Earlier work mainly focused on statistical and nonlinear features, while more recent studies have explored machine learning and deep learning methods to better capture temporal patterns in HRV signals [8,15,16,17]. However, most of these studies have been conducted in controlled environments, which makes it difficult to apply their findings directly to real-world settings such as surgical environments.

At the same time, wearable sensing technologies have made it possible to continuously monitor physiological signals in everyday [4,7,18]. While this creates new opportunities for stress classification, it also introduces challenges related to noisy data and reliable feature extraction. Another important issue is the lack of accurate ground truth labels, as self-reported measures are often subjective and do not capture rapid changes in stress. To overcome this, weak supervision and pseudo-labeling approaches have been proposed, where labels are generated using heuristic rules or proxy signals [19,20,21], although this may introduce some level of noise.

In addition, ensuring that models generalize well across individuals remains challenging due to inter-subject variability. LOSO cross-validation is commonly used to evaluate subject-independent performance [22,23], while random splits may lead to overly optimistic results. Despite these efforts, only limited work has combined personalized HRV feature extraction, weak supervision, and sequence-based deep learning in a single framework. Therefore, this study proposes an end-to-end pipeline that integrates these components and evaluates performance under both subject-dependent and subject-independent settings.

## 2. Materials and Methods

### 2.1. Ethical Approval

The ethical approval from Health Research Authority (HRA) was obtained, HRA reference 291694.

### 2.2. Participants

Seven consultant surgeons at the University Hospitals Coventry & Warwickshire NHS Trust (UHCW) performed 27 colorectal surgical procedures. All participating surgeons were consultants within a similar age group, providing a relatively homogeneous cohort in terms of clinical experience. The participants were healthy, with no history of any psychiatric conditions, were not taking any β-blockers, and anti-sympathetic medications, were non-smokers, had a BMI within the range of 18–35 kg/m^2^ and were able to provide informed consent. Although the surgical procedures varied in duration, resulting in recordings of different lengths, the same pre-operative baseline acquisition protocol was applied before every procedure to provide a consistent participant-specific physiological reference. A summary of the collected dataset, including the number of surgeries, the number of surgeons included, and the recording durations for each surgeon, is presented in Table 1.

### 2.3. Data Collection

**Figure 1:**
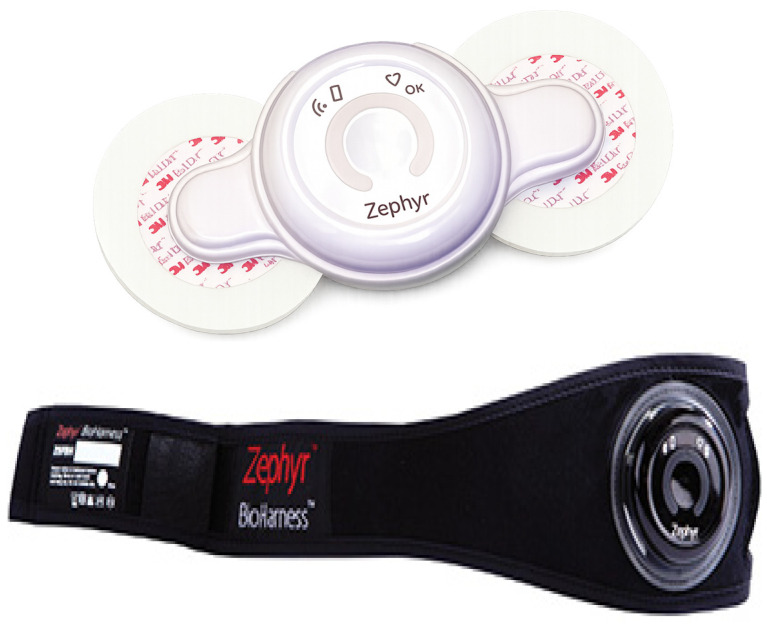
Zephyr BioPatch™ (**Upper**) and Zephyr BioPatch™ Side Strap (**Lower**).

Physiological data were recorded using Zephyr BioPatch devices (Zephyr Technology, Annapolis, MD, USA), a wearable HR monitor capable of continuous beat-to-beat HRV capture. The strap configuration was used, worn under the left chest and concealed beneath surgical clothing (Figure 1). The device was applied approximately two hours before surgery and removed immediately after completion. One of the authors (ZR) was present in the operating theatre to observe the cases and manually provide time-stamped notes for analysis. The observer was responsible only for annotating surgical events and did not have access to the HRV recordings or the derived surrogate labels during data collection.

### 2.4. Data Analysis

In general, HRV decreases in response to stress: lower HRV indicates higher stress levels, while higher HRV is associated with a more relaxed state. Although standard HRV analyses often use 5-min windows, studies support 1-min time-domain HRV segments for short-term HRV-based stress-state classification. This supports our use of per-minute chunks to reflect rapid changes in autonomic state as well as maximising the amount of usable data [9,24].

Moreover, recent research demonstrates that specific HRV indices obtained from 10–30 s time frames show reliability comparable to that of short-term HRV indices generated from traditional recording lengths of 5 min or more. This applies exclusively to specific categories of HRV measures, particularly time-domain indices, rather than frequency-domain indices, which require a minimum period of one minute [9,24,25]. In addition, the use of the mean SDNN is well established in the literature, supporting its inclusion alongside the outcomes of the clustering step, as will be presented in Section 3 [5,26].

### 2.5. Data Acquisition and Preprocessing

The HRV data were structured into minute-by-minute CSV files, one for the baseline phase (pre-scrubbing) and one for the surgery phase. Filenames encoded participant ID, session number, and phase, which were extracted programmatically using the filenames. The dataset consists of timestamped HRV measurements for each participant, capturing physiological responses over time. To improve data quality, implausible HRV values were removed using a predefined physiological range of 0–200 ms. The cleaned time-series data were then segmented into non-overlapping one-minute intervals, with each interval treated as a single observation. This aggregation helps reduce high-frequency noise while preserving meaningful temporal structure, enabling consistent analysis across subjects [25].

### 2.6. Feature Extraction and Normalization

For each one-minute interval, a set of statistical features was computed to summarise HRV dynamics. These features were selected to capture different aspects of the signal, including central tendency, variability, distribution shape, and complexity. Specifically, mean and median were used to represent central tendency, while variability was characterised using standard deviation, interquartile range, coefficient of variation, and median absolute deviation. Minimum and maximum values captured extrema, whereas skewness and kurtosis described distribution shape. Entropy was included to reflect signal complexity. To account for inter-subject physiological variability, HRV-derived features were normalised using surgeon-specific min–max scaling. To avoid information leakage, preprocessing was performed separately within each training fold. For each fold, missing values were imputed using the median of the training subset, and feature scaling parameters were estimated from the training subset only. The fitted imputer and scaler were then applied unchanged to the corresponding validation and held-out test data.

### 2.7. Stress Label Generation

Stress labels were generated using a subject-specific thresholding strategy based on the extracted SDNN. For each participant, the baseline files were merged, and the mean baseline for SDNN was calculated. This value was used as an individual threshold, whereby observations with HRV values below the threshold were labelled as stress and those above the threshold were labelled as non-stress. This allowed the labelling process to adapt to individual physiological baselines while assigning a binary stress label to each observation. As these labels are not based on external annotations or validated clinical assessments, they are treated as surrogate (weak) labels. This approach allows for scalable labelling while maintaining a degree of personalisation across subjects. To accommodate inter-individual differences in HRV, a participant-specific approach was adopted for both normalization and threshold generation. Features were scaled using each surgeon’s HRV range, and surrogate stress labels were derived from their individual baseline recordings, allowing stress classification to be tailored to each participant’s physiological characteristics.

The generated labels should be interpreted as surrogate indicators of HRV-derived physiological states rather than direct measurements of psychological stress. They were introduced to provide a practical supervisory signal in the absence of continuous, independently validated stress annotations during surgery. Although the machine learning models were trained using statistical features derived from the HRV signal rather than the SDNN threshold itself, the learned patterns remain linked to the adopted surrogate labelling strategy. Therefore, the proposed framework should be interpreted as classifying HRV-derived surrogate physiological states rather than independently validated psychological stress.

### 2.8. Unsupervised Analysis

**Figure 2:**
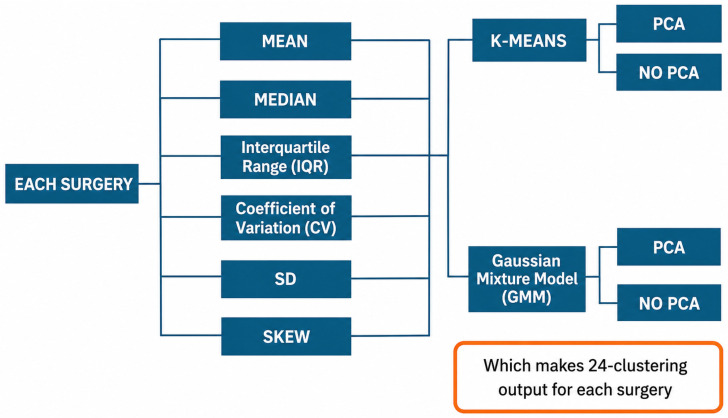
Unsupervised analysis flowchart.

To better understand the structure of the feature space, unsupervised learning methods were applied. K-means clustering was used to group observations based on feature similarity, while Gaussian Mixture Models (GMM) provided a probabilistic perspective on cluster membership. For both methods, the number of clusters was fixed at k=2, consistent with the binary HRV-derived surrogate labelling framework used throughout the study. During method development, alternative cluster numbers were explored using elbow and silhouette analyses. However, because the objective of the clustering analysis was to assess whether unsupervised grouping broadly aligned with the predefined binary surrogate labels, a two-cluster solution was considered the most appropriate and interpretable configuration. Principal Component Analysis (PCA) was also evaluated as a dimensionality reduction step, with the number of principal components selected to retain 95% of the total variance. Clustering was performed both on the original feature set and on the PCA-transformed features. The resulting clusters were compared qualitatively with the generated stress labels to explore the extent to which data-driven groupings align with the heuristic labelling strategy. To provide a quantitative assessment, cluster assignments were also compared with the surrogate stress labels, and accuracy, F1-score, sensitivity, and specificity were calculated to measure the level of agreement between the clustering output and the threshold-based labelling approach. The overall workflow of the unsupervised analysis is illustrated in Figure 2.

It should be noted that the clustering analysis was not intended to independently validate the personalised HRV-derived labels. Rather, it was performed as an exploratory analysis to investigate whether unsupervised clustering of HRV features produced physiological groupings that broadly aligned with the personalised labelling strategy. Because both approaches were derived from HRV measurements, the clustering results should be interpreted as an assessment of internal consistency rather than validation against an independent ground truth.

### 2.9. Sequence Construction

To capture temporal dependencies in HRV signals, the data were further transformed into sequential inputs using a sliding window approach. Each sample consisted of a sequence of consecutive one-minute feature vectors, allowing the models to incorporate temporal context into their predictions. Multiple sequence lengths were considered, including 1, 2, 5, 10, and 20 min, in order to evaluate the impact of temporal resolution on model performance. Each sequence was assigned the label corresponding to its final time step, resulting in a sequence-to-one classification framework.

To avoid temporal information leakage, sequence construction was performed after data partitioning. For each validation strategy, the training, validation, and test sets were created before the sliding-window procedure was applied. Consequently, sequences were generated independently within each subset, ensuring that no overlapping temporal windows were shared between training, validation, and testing data.

### 2.10. Deep Learning Models

Two deep learning architectures were investigated for modelling temporal patterns in the HRV data: a CNN and a LSTM network. The CNN was selected because of its ability to identify local temporal patterns within a sequence, whereas the LSTM was included to capture temporal dependencies over longer periods.

The CNN model consisted of two one-dimensional convolutional layers with 32 and 64 filters, respectively, both using a kernel size of three and ReLU activation functions. A dropout rate of 0.2 was applied after each convolutional layer. The output was then passed through an adaptive average pooling layer and two fully connected layers before generating the final binary prediction. The LSTM architecture comprised a single recurrent layer with 64 hidden units, followed by two fully connected layers. ReLU activation and a dropout rate of 0.2 were applied before the final output layer.

Both models were trained using the Adam optimiser (learning rate = 0.001, weight decay = 0.0001) and a batch size of 128. Training was limited to a maximum of 25 epochs. Binary cross-entropy with logits was used as the loss function, and class weights were applied to reduce the impact of class imbalance. To minimise overfitting, early stopping based on validation area under the curve (AUC) was employed, with training halted after five epochs without improvement. For each fold, 20% of the training data were used as a validation set. ROC-AUC was calculated using the continuous prediction probabilities generated by the model, whereas accuracy, F1-score, sensitivity, and specificity were calculated after converting these probabilities into binary class predictions using a threshold of 0.5.

The network hyperparameters were selected based on commonly adopted configurations reported in previous studies of physiological time-series classification and wearable sensor analysis, together with preliminary experiments to ensure stable convergence while limiting model complexity [27,28]. Specifically, 32 convolutional filters and 64 LSTM hidden units were chosen to provide sufficient representational capacity without substantially increasing the risk of overfitting given the available dataset size. A dropout rate of 0.2 was applied as a regularisation strategy, while the Adam optimiser was selected because of its widespread use and robust convergence behaviour in deep learning applications [29,30]. Training was performed for a maximum of 25 epochs with early stopping to prevent unnecessary optimisation once validation performance no longer improved [31].

For each training fold, an independent validation subset (20% of the training data) was randomly generated from the training data only and used to monitor validation performance for early stopping. The held-out test subject was never included in either the training or validation subsets. Consequently, early stopping decisions were based exclusively on the training-fold data, preventing information leakage into the test set.

The same hyperparameter settings were retained across all experiments to allow a direct comparison between sequence lengths and evaluation strategies. Sequence lengths of 1, 2, 5, 10, and 20 min were evaluated. All models were implemented in Python version 3.10.18 using the PyTorch 2.2.2 deep learning framework, and a random seed of 42 was used to improve reproducibility. Model training was performed on a MacBook Pro equipped with an Apple M1 chip.

### 2.11. Evaluation Strategy

Model performance was evaluated using two complementary validation strategies. First, a LOSO approach was employed, in which data from one participant were held out for testing while the models were trained on the remaining subjects. This process was repeated for all participants, providing a robust estimate of generalisation to unseen individuals. Second, a random train–test split was performed by pooling all data and dividing it into 70% training and 30% testing sets. While this approach provides an upper-bound estimate of performance, it does not account for inter-subject variability and is therefore interpreted with caution. All preprocessing parameters were estimated using the training data within each fold, and no information from the held-out participant was used during imputation or scaling. Sequence generation was also performed independently within each fold after data partitioning, preventing overlapping temporal windows from appearing simultaneously in the training and evaluation sets.

### 2.12. Performance Metrics

The performance of the models was assessed using a range of standard classification metrics, including accuracy, precision, recall, and F1-score. Accuracy measures the proportion of correctly classified observations, precision reflects the proportion of predicted stress instances that were correctly identified, recall (sensitivity) measures the proportion of actual stress instances correctly detected, and the F1-score provides a harmonic mean of precision and recall. In addition, the AUC and specificity were computed to provide a more comprehensive evaluation, particularly in the presence of potential class imbalance.

### 2.13. Statistical Analysis

Statistical analyses were performed using IBM SPSS Statistics for Mac, Version 29.0.2.0 (IBM Corp., Armonk, NY, USA). Since the performance measures were obtained from the same seven participants under multiple experimental conditions, non-parametric tests were used. The effect of sequence length (1, 2, 5, 10, and 20 min) on model performance was evaluated separately for the CNN and LSTM models using the Friedman test based on the leave-one-subject-out (LOSO) F1-scores. Pairwise comparisons between the CNN and LSTM models at each sequence length were performed using the Wilcoxon signed-rank test. The Wilcoxon results were interpreted using Holm’s sequential correction to account for multiple comparisons. Statistical significance was set at *p* < 0.05.

The 10 min sequence length was selected because it was among the most frequently occurring top-performing configurations across surgeons while maintaining strong overall performance. To evaluate robustness to random initialization, the representative 10 min CNN and LSTM models were retrained using five different random seeds under the same subject-wise LOSO protocol. The mean F1-score, AUC, training time, and number of trainable parameters were subsequently reported.

## 3. Results

This section presents the main findings from the proposed HRV-based surrogate stress-like physiological state classification pipeline. First, the unsupervised clustering analysis results are presented to examine how well different HRV features separate physiological states and to identify the useful feature groups. Second, the performance of sequence-based CNN and LSTM models is presented across different temporal window lengths.

### 3.1. Unsupervised Clustering Analysis

The summary of the clustering results for all surgeons is presented in Table 2. Clustering performance metrics were calculated by comparing the cluster assignments with the generated surrogate stress labels. To explore which features were most informative, the top four clustering outcomes were selected for each surgeon, regardless of the clustering method used.

Across all top-performing configurations (n = 28), mean- and median-based HRV SDNN values were equally prevalent, each appearing in 11 of 28 configurations (39.3%). Variability-based features were less common, with standard deviation (SD), interquartile range (IQR), and coefficient of variation (CV) appearing in only 6 of 28 configurations combined (21.4%). No top-performing configuration was based on HRV skewness. These findings are consistent with previous studies, where mean HRV metrics are often reported as stable and informative indicators in stress-related analysis. Based on this observation, mean-based SDNN were prioritised in the subsequent modelling experiments.

In addition, k-means clustering was more frequently associated with the top-performing configurations across surgeons, whereas GMM appeared only in a limited number of cases. This suggests that k-means provides more stable clustering behaviour for the SDNN used in this study. For detailed tables, see Supplementary Tables S1–S7.

### 3.2. Sequence Construction

The performance of different sequence lengths and deep learning models is summarised in Table 3, which reports mean performance across the seven LOSO folds. The dataset comprised 5160 stress observations (68.7%) and 2346 non-stress observations (31.3%), indicating a relatively balanced class distribution. Overall, model performance was broadly comparable across sequence lengths. Based on the mean LOSO results, the CNN model with a 10-min sequence length achieved the highest average performance (F1 = 0.831 ± 0.144), although performance was broadly comparable across all sequence lengths. Similar performance was observed for the LSTM model, although with greater variability across subjects (F1 = 0.824 ± 0.164). Sensitivity was generally higher than specificity across both models, indicating that stress observations were more readily identified than non-stress observations. While shorter sequence lengths produced the highest average performance in Table 3, and the surgeon-level analysis which will be presented later, showed that longer sequence lengths of 10–20 min were more frequently associated with the top-performing models. Extra detailed results are provided in Supplementary Tables S8 and S9.

Friedman tests were performed to evaluate the effect of sequence length on LOSO F1-score performance. No significant effect of sequence length was observed for either the CNN model (χ^2^(4) = 7.429, *p* = 0.115) or the LSTM model (χ^2^(4) = 5.943, *p* = 0.203), indicating consistent performance across the evaluated sequence lengths.

Wilcoxon signed-rank tests were then used to compare the CNN and LSTM models at each sequence length. No significant differences were observed at the 1, 2, 5, or 10 min sequence lengths (*p* = 0.499, 0.735, 0.398, and 0.176, respectively). Although the comparison at 20 min was nominally significant (Z = −2.366, *p* = 0.018), it did not remain significant after Holm’s sequential correction. Overall, neither sequence length nor model architecture produced statistically significant differences in LOSO F1-score performance, as summarized in Table 4.

To evaluate robustness to random initialization, the representative 10 min CNN and LSTM models were retrained using five independent random seeds under the same subject-wise LOSO validation protocol. As shown in Table 5, both models demonstrated stable performance across different random initializations, with only minor variations in F1-score and AUC. Although the LSTM contained more trainable parameters than the CNN (19,265 vs. 8449), it required a shorter average training time (96.8 ± 4.4 s vs. 154.2 ± 3.9 s). These findings indicate that both models are robust to random initialization while maintaining computational efficiency.

It should be noted that ROC-AUC reflects the model’s ability to rank positive and negative samples across all possible decision thresholds, whereas accuracy, F1-score, sensitivity, and specificity depend on the fixed classification threshold. Consequently, perfect AUC values may occur despite lower threshold-dependent metrics when the predicted probabilities correctly rank the samples but a small number of observations are misclassified after thresholding.

In the pooled 70/30 split evaluation, performance remained broadly comparable across all sequence lengths. Shorter temporal windows generally achieved the highest average performance, while increasing the sequence length did not consistently improve model performance. Similarly, under LOSO validation, no consistent advantage was observed for either shorter or longer temporal windows across all held-out participants. Although some individual surgeons achieved their best performance using longer sequence lengths, others performed equally well or better with shorter windows. These observations suggest that the optimal temporal context may vary between individuals and should therefore be interpreted as descriptive trends within the present dataset rather than statistically significant performance differences.

As shown in Table 6, the 5 and 10-min windows appeared most often among the top-performing configurations, while 20-min windows also featured prominently. Although shorter sequence lengths produced strong results for some surgeons, the overall trend suggests that incorporating a longer temporal context may improve the ability of CNN and LSTM models to capture stress-related patterns in HRV. These findings indicate that stress-related physiological responses may develop over several minutes and that longer observation windows can provide additional information for classification.

In terms of model architecture, CNN and LSTM models showed comparable performance overall, with no consistent advantage observed for either model across all configurations. Interestingly, performance under LOSO validation was consistently higher than under the pooled (70/30) split. Although the LOSO results indicate encouraging cross-subject performance within this dataset, they should not be interpreted as definitive evidence of strong generalisation because the cohort was relatively small and the observed differences may partly reflect characteristics of the dataset and evaluation procedure. However, this behaviour may also reflect differences in data distribution between the two evaluation strategies, such as class balance or the structure of the generated labels. See Supplementary Materials (Tables S11–S17).

This observation highlights the importance of carefully selecting validation strategies when evaluating physiological machine learning models, as different approaches may lead to different conclusions about model performance. Detailed results for each surgeon and are provided in the Supplementary Materials Tables S10–S17.

Across all surgeons, LSTM with a 10-min sequence length and CNN with a 10-min sequence length were the most frequently occurring top-performing configurations, each appearing three times among the best or second-best models. LSTM with 20-min and 2-min sequence lengths, together with CNN using 20-min sequences, also appeared repeatedly, suggesting that no single architecture or sequence length consistently outperformed all others across surgeons.

## 4. Discussion

This study explored the use of HRV-based features for stress and workload classification in surgical settings, with a particular focus on personalised modelling, temporal dynamics, and evaluation strategy. Overall, the findings provide useful insight into both the behaviour of SDNN and the performance of sequence-based models in this context.

One of the clearest observations from the clustering analysis was that, within the present dataset mean-based SDNN generally provided more consistent separation than the other HRV-derived labelling strategies. This is broadly in line with established HRV literature, including the Task Force document on Heart Rate Variability (1996) and the review by Shaffer and Ginsberg, which emphasise the robustness and interpretability of time-domain HRV measures [5,6]. It is also consistent with broader physiological frameworks, such as the neurovisceral integration model proposed by Thayer and Lane and the concept of allostatic load described by McEwen, both of which link autonomic regulation to stress and cognitive function [32]. In practical terms, this result is not entirely surprising. In real surgical environments, where recordings are often affected by motion artefacts and external noise, more stable features such as the mean are likely to be more reliable than higher-order variability measures.

In addition, the clustering results showed that k-means was more frequently associated with top-performing configurations than to GMM, suggesting greater stability for the feature space considered. This behaviour can be explained by the nature of the extracted SDNN, which are primarily statistical summaries and tend to form relatively compact and well-separated clusters. In such cases, k-means performs effectively due to its assumption of spherical cluster structure. In contrast, GMM provides greater flexibility by modelling complex and overlapping distributions, but this also makes it more sensitive to noise and data variability. Similar observations have been reported in comparative studies, where k-means performs well on well-separated and low-dimensional feature spaces, while GMM is more advantageous for complex or overlapping data distributions [33,34]. This suggests that, for HRV-based stress analysis, simpler clustering approaches such as k-means may offer more robust and consistent performance.

It is important to interpret these clustering results within the context of the proposed surrogate labelling framework. Because both the clustering analysis and the personalised labels were derived from HRV measurements, agreement between the two should not be interpreted as an independent validation of stress classification. Instead, the clustering analysis served as an exploratory assessment of whether unsupervised physiological groupings were broadly consistent with the personalised HRV-derived surrogate labelling strategy. Therefore, these findings support the internal consistency of the proposed framework rather than providing validation against an external ground truth.

Another interesting finding relates to the influence of sequence length on model performance. Although high classification performance was achieved across all evaluated temporal windows, some of the highest-performing individual configurations were associated with sequence lengths of 10–20 min. However, this trend was not consistent across all held-out surgeons and therefore should be interpreted as a descriptive observation rather than definitive evidence that longer temporal windows are superior. This suggests that incorporating a broader temporal context may help capture stress-related changes in HRV that develop gradually over time. While previous studies have shown that ultra-short HRV recordings can provide useful information for stress assessment [9,24,25], the present findings indicate that, within this dataset, longer observation windows often provided additional discriminatory information. Similar observations have been reported in wearable physiological monitoring studies, where temporal context plays an important role in identifying changes in autonomic activity [15,30]. One possible explanation is that stress responses are dynamic processes that evolve over several minutes, allowing longer sequences to capture patterns that may not be fully represented within shorter windows. These findings suggest that the choice of sequence length should be carefully considered when designing HRV-based stress classification systems, as longer temporal windows may offer advantages for detecting subtle physiological changes. See Supplementary Materials (Tables S11–S17).

Although the aggregate LOSO results and the surgeon-specific best-performing models may appear inconsistent, these analyses address different questions. The aggregate results summarise the average performance across all surgeons, whereas the surgeon-level analysis identifies the best-performing configuration for each individual surgeon. Consequently, although shorter sequence lengths may achieve slightly higher average performance across the cohort, longer sequence lengths were more frequently selected as the best-performing configurations for individual surgeons. This observation further highlights the inter-individual variability in HRV responses and suggests that the optimal temporal context may differ between surgeons.

When considering model architecture, CNN and LSTM models showed broadly comparable performance, with no clear or consistent advantage for either approach. This is in line with recent reviews, including those by Khandelwal and Sharma as well as Hoang, which note that both convolutional and recurrent models can effectively capture temporal patterns in physiological data [16,17]. In this case, the relatively short sequence lengths may explain why more complex temporal modelling did not provide a clear benefit. It appears that local temporal structure, which can be captured effectively by CNNs, is sufficient for this task. Although some model configurations achieved near-perfect AUC values, sensitivity and specificity were not consistently balanced across all surgeons and experimental settings. In several cases, high overall discrimination performance was accompanied by lower sensitivity or specificity, suggesting that the models did not perform equally well across both classes. This variability is likely related to differences in individual physiological responses, dataset size, and the distribution of the surrogate labels.

From a practical perspective, the comparable performance of CNN and LSTM models suggests that the simpler CNN architecture may be preferable for real-time stress monitoring applications. CNNs generally require fewer sequential computations than LSTMs, resulting in lower computational complexity and faster inference, which may facilitate deployment in resource-constrained or real-time clinical environments such as the operating room. Nevertheless, the choice of architecture should ultimately depend on the specific application requirements and available computational resources [35].

An additional robustness analysis showed that retraining the representative 10-min CNN and LSTM models using five different random seeds produced only minor variations in F1-score and AUC, indicating that the reported performance was stable with respect to random initialization. Perhaps the most unexpected result in this study is that performance under LOSO validation was higher than under the pooled (70/30) split. This contrasts with much of the existing literature. For example, work by Saeb et al. highlights how random splits often lead to overly optimistic performance estimates due to subject-specific patterns being shared between training and testing data [11]. Similarly, the TRIPOD + AI guidelines by Collins et al. emphasise the importance of subject-wise validation when assessing generalisation [12].

In the present case, however, the opposite trend was observed. One possible explanation for this observation is the personalized threshold-based labelling strategy adopted in this study, which aligns surrogate labels with each surgeon’s individual physiological baseline and may reduce inter-subject variability. However, this interpretation should be regarded as a hypothesis rather than a definitive explanation. Other factors, including the relatively small sample size, variability in subject-specific data distributions, differences in fold composition, and statistical variability associated with cross-validation, may also have contributed to the observed performance. Therefore, further evaluation using larger and more diverse datasets is required before drawing firm conclusions regarding the influence of personalized labelling on model generalization. Importantly, no data from the held-out test subject were used during model training under the LOSO framework. At the same time, this result should be interpreted with some caution. As class distributions were comparable across the two evaluation strategies, differences in class balance are unlikely to explain the observed outcome. Similar concerns have been raised in studies of cross-subject evaluation, where performance can vary depending on how data are structured and split [22,23].

Taken together, these observations reinforce the importance of carefully selecting and interpreting evaluation strategies in physiological machine learning. As noted in prior work, choice of validation method can significantly influence reported results. By reporting both pooled and LOSO evaluations, this study provides a more complete and transparent view of model performance.

Despite these encouraging findings, several limitations should be acknowledged. First, the stress labels used in this study are surrogate labels derived from HRV-based thresholds, rather than externally validated ground truth such as expert annotation or synchronised subjective reports. While pseudo-labelling approaches are increasingly used in settings where annotation is difficult, as discussed by Ran et al. and Kage et al., they may introduce noise and limit interpretability [19,21]. Second, the dataset is relatively small and includes a limited number of surgeons, which may increase the risk of model overfitting, contribute to variability in the reported performance metrics, and restrict the generalisability of the findings. Finally, physiological data collected in real surgical environments are inherently noisy, which may affect both feature quality and model performance.

Future work should address several limitations of the present framework. In particular, the proposed surrogate labels were derived from participant-specific SDNN thresholds and were not validated against an independent measure of psychological stress. Consequently, the reported performance reflects the classification of HRV-derived surrogate stress-like physiological states rather than clinically validated stress. Future studies should therefore incorporate independent reference measures, such as validated psychological questionnaires, behavioral observations, expert annotations, or biochemical stress markers, to improve label quality and strengthen model validation. Evaluating alternative personalised thresholding strategies may also help assess the robustness of the proposed surrogate labelling framework. Expanding the dataset to include a larger number of participants and procedures would also improve the robustness and generalisability of the findings. In addition, exploring more advanced modelling approaches, such as attention-based or transformer-based architectures, and integrating multimodal physiological signals may provide further insight into temporal patterns associated with stress-related physiological responses.

## 5. Conclusions

This study presented an end-to-end pipeline for HRV-based classification of HRV-derived surrogate stress-like physiological states in surgical environments, integrating personalised feature extraction, surrogate label generation, and sequence-based modelling. By combining physiological signal processing with machine learning techniques, the proposed framework demonstrates the feasibility of classifying HRV-derived surrogate physiological states using personalised machine learning from wearable HRV data.

Within the present dataset, the findings demonstrate that mean-based SDNN are particularly effective for distinguishing physiological states. Analysis of sequence length showed that the highest-performing models were most frequently associated with longer temporal windows of 10–20 min, suggesting that incorporating a broader temporal context may improve the detection of stress-related physiological patterns. In addition, CNN and LSTM models showed comparable performance, indicating that model architecture may be less critical than feature representation and data quality in this context.

An interesting observation was that LOSO validation produced performance comparable to, and in some cases higher than, pooled random splits. Although personalised surrogate labelling may have contributed to this behaviour, the relatively small cohort and dataset characteristics mean that this interpretation remains hypothetical and requires confirmation in larger independent studies.

Despite these promising results, several limitations remain, including the use of surrogate labels and the relatively small dataset size. Future work should focus on incorporating multi-modal data sources, expanding the dataset, and exploring more advanced modelling approaches to further improve robustness and interpretability.

In summary, this study represents a proof of concept demonstrating the feasibility of personalised machine learning for the classification of HRV-derived surrogate stress-like physiological states in surgical environments. By emphasising personalised modelling, extended temporal analysis, and rigorous evaluation, it provides a foundation for future research in physiological monitoring and clinical performance assessment. However, further validation using larger and more diverse cohorts, together with independent measures of psychological stress, is required before the proposed framework can be considered for routine clinical deployment.

## Figures and Tables

**Table 1:** Summary of the collected data.

Code	No. of Surgeries	Total Duration of Recordings (h)	Total Duration of Surgery Recording (h)	Total Duration of Baseline Recording (h)
A	6	26.8	19.7	7.1
B	6	27.5	20	7.5
C	5	21.88	20.75	1.13
D	5	20.3	12.8	7.5
E	2	5.6	5.2	0.5
F	1	5.7	4.6	1.1
G	2	11.1	9.4	1.7
Total	27	118.98	92.45	26.53

**Table 2:** Summary of the clustering performance based on agreement between cluster assignments and surrogate stress labels.

Code	Mode	Label	Acc	F1	Sens	Spec
A	kMeans no pca	Median	0.789	0.726	0.694	0.919
A	kMeans pca 95	Mean	0.789	0.726	0.694	0.919
A	kMeans pca 95	Median	0.789	0.726	0.694	0.919
A	kMeans no pca	Mean	0.788	0.725	0.693	0.919
B	kMeans pca 95	Mean	0.933	0.957	0.930	0.968
B	kMeans no pca	Mean	0.932	0.956	0.930	0.965
B	kMeans pca 95	Median	0.929	0.955	0.923	0.976
B	kMeans no pca	Median	0.928	0.954	0.923	0.972
C	gmm no pca	Median	0.896	0.914	0.887	0.935
C	kMeans pca 95	Median	0.894	0.914	0.888	0.925
C	kMeans no pca	Median	0.893	0.913	0.886	0.925
C	gmm no pca	Mean	0.887	0.907	0.881	0.936
D	kMeans no pca	Mean	0.901	0.916	0.928	0.923
D	kMeans pca 95	Mean	0.901	0.916	0.928	0.924
D	kMeans pca 95	Median	0.900	0.917	0.920	0.931
D	kMeans no pca	Median	0.900	0.916	0.920	0.930
E	kMeans no pca	Median	0.950	0.964	0.970	0.920
E	kMeans pca 95	Median	0.950	0.964	0.970	0.920
E	gmm no pca	Median	0.947	0.962	0.970	0.911
E	kMeans no pca	Mean	0.944	0.959	0.974	0.898
F	kMeans no pca	Mean	0.930	0.927	1.000	0.875
F	kMeans pca 95	Mean	0.930	0.927	1.000	0.875
F	gmm no pca	Mean	0.927	0.924	0.993	0.875
F	kMeans no pca	Median	0.895	0.886	1.000	0.824
G	gmm pca 95	SD	0.768	0.845	0.779	0.710
G	gmm pca 95	IQR	0.759	0.841	0.769	0.693
G	gmm no pca	CV	0.701	0.771	0.734	0.634
G	gmm pca 95	Mean	0.677	0.786	0.722	0.361

**Table 3:** Performance of CNN and LSTM models using mean HRV under subject-wise LOSO validation. Results are reported as mean ± standard deviation across the seven held-out surgeons. The dataset comprised 5160 stress observations (68.7%) and 2346 non-stress observations (31.3%). Results are reported as the mean performance across LOSO folds.

Model	Sequence Length	F1-Score (Mean ± SD)	F1-Score 95% CI	AUC (Mean ± SD)	AUC 95% CI	Sensitivity (Mean ± SD)	Specificity (Mean ± SD)
CNN	1 min	0.780 ± 0.182	0.612–0.948	1.000 ± 0.000	1.000–1.000	0.700 ± 0.268	0.937 ± 0.117
LSTM	1 min	0.743 ± 0.335	0.433–1.000	1.000 ± 0.000	1.000–1.000	0.701 ± 0.366	0.881 ± 0.208
CNN	2 min	0.817 ± 0.159	0.670–0.964	0.999 ± 0.004	0.995–1.000	0.744 ± 0.245	0.946 ± 0.116
LSTM	2 min	0.829 ± 0.155	0.686–0.972	0.999 ± 0.004	0.995–1.000	0.756 ± 0.240	0.951 ± 0.095
CNN	5 min	0.821 ± 0.157	0.676–0.966	0.986 ± 0.018	0.969–1.000	0.750 ± 0.239	0.947 ± 0.098
LSTM	5 min	0.821 ± 0.168	0.666–0.976	0.993 ± 0.011	0.983–1.000	0.746 ± 0.250	0.950 ± 0.101
CNN	10 min	0.831 ± 0.144	0.698–0.964	0.986 ± 0.021	0.967–1.000	0.769 ± 0.229	0.929 ± 0.133
LSTM	10 min	0.824 ± 0.164	0.672–0.976	0.987 ± 0.015	0.973–1.000	0.751 ± 0.244	0.947 ± 0.119
CNN	20 min	0.827 ± 0.142	0.696–0.958	0.973 ± 0.010	0.964–0.982	0.766 ± 0.225	0.899 ± 0.155
LSTM	20 min	0.809 ± 0.167	0.655–0.963	0.983 ± 0.022	0.963–1.000	0.744 ± 0.253	0.906 ± 0.156

**Table 4:** Statistical comparisons of sequence lengths and model architectures based on LOSO F1-scores.

Comparison	Test	Statistic	*p*-Value	Conclusion
CNN across sequence lengths	Friedman	χ^2^(4) = 7.429	0.115	Not significant
LSTM across sequence lengths	Friedman	χ^2^(4) = 5.943	0.203	Not significant
CNN vs. LSTM (1 min)	Wilcoxon	Z = −0.676	0.499	Not significant
CNN vs. LSTM (2 min)	Wilcoxon	Z = −0.338	0.735	Not significant
CNN vs. LSTM (5 min)	Wilcoxon	Z = −0.845	0.398	Not significant
CNN vs. LSTM (10 min)	Wilcoxon	Z = −1.352	0.176	Not significant
CNN vs. LSTM (20 min)	Wilcoxon	Z = −2.366	0.018 ^†^	Not significant after Holm correction

^†^ Nominally significant before Holm’s sequential correction.

**Table 5:** Model robustness and computational performance under subject-wise LOSO validation.

Model	Trainable Parameters	Training Time (s)	F1-Score (Mean ± SD)	AUC (Mean ± SD)
CNN	8449	154.2 ± 3.9	0.826 ± 0.006	0.984 ± 0.003
LSTM	19,265	96.8 ± 4.4	0.829 ± 0.003	0.987 ± 0.001

**Table 6:** Summary of best and second-best models for each surgeon in LOSO.

Test_id	Rank	Model	Sequence Length	Acc	F1	Auc	Sensitivity	Specificity
A	1	LSTM	10	0.98	0.99	1.00	1.00	0.95
A	2	LSTM	5	0.97	0.98	1.00	1.00	0.92
B	1	CNN	10	0.87	0.91	0.99	0.84	1.00
B	2	LSTM	10	0.87	0.92	0.98	0.84	1.00
C	1	LSTM	2	0.94	0.95	1.00	0.91	1.00
C	2	CNN	10	0.93	0.94	0.99	0.89	0.99
D	1	LSTM	20	0.88	0.92	1.00	1.00	0.64
D	2	LSTM	10	0.89	0.92	1.00	1.00	0.68
E	1	CNN	20	0.69	0.70	0.96	0.54	1.00
E	2	CNN	10	0.68	0.69	0.94	0.53	1.00
F	1	CNN	20	0.90	0.87	0.98	0.78	0.99
F	2	CNN	10	0.87	0.83	0.99	0.71	1.00
G	1	LSTM	1	0.64	0.73	1.00	0.58	1.00
G	2	LSTM	2	0.49	0.58	1.00	0.41	1.00

## Data Availability

The datasets presented in this article are not readily available because the data are part of an ongoing study. Requests to access the datasets should be directed to Abdulaziz Almuhin.

## Supplementary Materials

The following supporting information can be downloaded at https://www.mdpi.com/article/10.3390/s26154822/s1. Table S1: A all surgeries clustering results pooled together; Table S2: B all surgeries clustering results pooled together; Table S3: C all surgeries clustering results pooled together; Table S4: D all surgeries clustering results pooled together; Table S5: E all surgeries clustering results pooled together; Table S6: F all surgeries clustering results pooled together; Table S7: G all surgeries clustering results pooled together; Table S8: Class distribution for each surgeon used in the subject-wise LOSO; Table S9. Class distribution across evaluation strategies; Table S10: Result with different sequence length and different models for 70\30; Table S11: Result with different sequence length and different models for LOSO/A; Table S12: Result with different sequence length and different models for LOSO/B; Table S13: Result with different sequence length and different models for LOSO/C; Table S14: Result with different sequence length and different models for LOSO/D; Table S15: Result with different sequence length and different models for LOSO/E; Table S16: Result with different sequence length and different models for LOSO/F; Table S17: Result with different sequence length and different models for LOSO/G.

## Abbreviations

The following abbreviations are used in this manuscript:

HRV	Heart Rate Variability
CNN	Convolutional Neural Networks
LSTM	Long Short-Term Memory
LOSO	Leave-One-Subject-Out
HRA	Health Research Authority
UHCW	University Hospitals Coventry & Warwickshire NHS Trust
SDNN	Standard Deviation of Normal-to-Normal intervals
GMM	Gaussian Mixture Models
PCA	Principal Component Analysis
AUC	Area Under the Curve
