# Supplementary material for: HRV-Based Surrogate Classification in Surgical Environments Using Personalised Labelling and Sequence Modelling
Source: Sensors (Basel). 2026 Jul 30;26(15):4822. doi: 10.3390/s26154822 (PMC13469991; doi:10.3390/s26154822)
Supplement: Supplementary file 1 [file sensors-26-04822-s001.zip › sensors-4416111-supplementary.pdf]

Mode	Label	Acc	F1	Sens	Spec
gmm_no_pca	stress_cv	0.614	0.619	0.590	0.564
gmm_no_pca	stress_iqr	0.626	0.715	0.690	0.458
gmm_no_pca	stress_mean	0.711	0.681	0.671	0.745
gmm_no_pca	stress_median	0.712	0.682	0.671	0.745
gmm_no_pca	stress_sd	0.650	0.734	0.697	0.505
gmm_no_pca	stress_skew	0.528	0.553	0.580	0.461
gmm_pca_95	stress_cv	0.646	0.697	0.788	0.371
gmm_pca_95	stress_iqr	0.732	0.821	0.887	0.308
gmm_pca_95	stress_mean	0.750	0.705	0.773	0.491
gmm_pca_95	stress_median	0.757	0.715	0.785	0.501
gmm_pca_95	stress_sd	0.773	0.848	0.900	0.371
gmm_pca_95	stress_skew	0.527	0.602	0.735	0.276
kmeans_no_pca	stress_cv	0.603	0.593	0.525	0.637
kmeans_no_pca	stress_iqr	0.613	0.702	0.663	0.498
kmeans_no_pca	stress_mean	0.788	0.725	0.693	0.919
kmeans_no_pca	stress_median	0.789	0.726	0.694	0.919
kmeans_no_pca	stress_sd	0.629	0.718	0.666	0.535
kmeans_no_pca	stress_skew	0.529	0.538	0.538	0.509
kmeans_pca_95	stress_cv	0.602	0.592	0.524	0.637
kmeans_pca_95	stress_iqr	0.613	0.703	0.663	0.498
kmeans_pca_95	stress_mean	0.789	0.726	0.694	0.919
kmeans_pca_95	stress_median	0.789	0.726	0.694	0.919
kmeans_pca_95	stress_sd	0.630	0.718	0.667	0.535
kmeans_pca_95	stress_skew	0.529	0.538	0.539	0.509

Table S1: A all surgeries clustering results pooled together

Mode	Label	Acc	F1	Sens	Spec
gmm_no_pca	stress_cv	0.566	0.645	0.698	0.355
gmm_no_pca	stress_iqr	0.695	0.791	0.804	0.411
gmm_no_pca	stress_mean	0.881	0.925	0.900	0.806
gmm_no_pca	stress_median	0.880	0.925	0.895	0.814
gmm_no_pca	stress_sd	0.735	0.824	0.811	0.481
gmm_no_pca	stress_skew	0.545	0.422	0.390	0.683
gmm_pca_95	stress_cv	0.696	0.774	0.881	0.424
gmm_pca_95	stress_iqr	0.835	0.883	0.904	0.662
gmm_pca_95	stress_mean	0.773	0.849	0.829	0.567
gmm_pca_95	stress_median	0.775	0.850	0.827	0.575
gmm_pca_95	stress_sd	0.873	0.913	0.903	0.780
gmm_pca_95	stress_skew	0.519	0.373	0.344	0.673
kmeans_no_pca	stress_cv	0.545	0.629	0.678	0.331
kmeans_no_pca	stress_iqr	0.698	0.793	0.805	0.421
kmeans_no_pca	stress_mean	0.932	0.956	0.930	0.965
kmeans_no_pca	stress_median	0.928	0.954	0.923	0.972
kmeans_no_pca	stress_sd	0.743	0.829	0.815	0.502
kmeans_no_pca	stress_skew	0.545	0.372	0.289	0.771
kmeans_pca_95	stress_cv	0.544	0.628	0.676	0.331

kmeans_pca_95	stress_iqr	0.697	0.792	0.804	0.421
kmeans_pca_95	stress_mean	0.933	0.957	0.930	0.968
kmeans_pca_95	stress_median	0.929	0.955	0.923	0.976
kmeans_pca_95	stress_sd	0.742	0.829	0.814	0.502
kmeans_pca_95	stress_skew	0.546	0.374	0.291	0.771

Table S2: B all surgeries clustering results pooled together

Mode	Label	Acc	F1	Sens	Spec
gmm_no_pca	stress_cv	0.582	0.678	0.678	0.388
gmm_no_pca	stress_iqr	0.566	0.627	0.613	0.460
gmm_no_pca	stress_mean	0.887	0.907	0.881	0.936
gmm_no_pca	stress_median	0.896	0.914	0.887	0.935
gmm_no_pca	stress_sd	0.580	0.647	0.614	0.475
gmm_no_pca	stress_skew	0.541	0.410	0.375	0.667
gmm_pca_95	stress_cv	0.795	0.859	0.953	0.519
gmm_pca_95	stress_iqr	0.790	0.852	0.973	0.501
gmm_pca_95	stress_mean	0.656	0.690	0.727	0.373
gmm_pca_95	stress_median	0.655	0.693	0.732	0.377
gmm_pca_95	stress_sd	0.839	0.890	0.980	0.581
gmm_pca_95	stress_skew	0.550	0.281	0.217	0.799
kmeans_no_pca	stress_cv	0.579	0.678	0.682	0.371
kmeans_no_pca	stress_iqr	0.562	0.624	0.612	0.450
kmeans_no_pca	stress_mean	0.886	0.908	0.882	0.928
kmeans_no_pca	stress_median	0.893	0.913	0.886	0.925
kmeans_no_pca	stress_sd	0.580	0.647	0.615	0.468
kmeans_no_pca	stress_skew	0.540	0.416	0.393	0.652
kmeans_pca_95	stress_cv	0.580	0.679	0.682	0.374
kmeans_pca_95	stress_iqr	0.561	0.624	0.612	0.448
kmeans_pca_95	stress_mean	0.885	0.907	0.882	0.927
kmeans_pca_95	stress_median	0.894	0.914	0.888	0.925
kmeans_pca_95	stress_sd	0.579	0.647	0.615	0.466
kmeans_pca_95	stress_skew	0.539	0.415	0.393	0.651

Table S3: C all surgeries clustering results pooled together

Mode	Label	Acc	F1	Sens	Spec
gmm_no_pca	stress_cv	0.605	0.674	0.584	0.602
gmm_no_pca	stress_iqr	0.617	0.713	0.664	0.504
gmm_no_pca	stress_mean	0.795	0.821	0.844	0.846
gmm_no_pca	stress_median	0.791	0.819	0.834	0.849
gmm_no_pca	stress_sd	0.625	0.728	0.658	0.507
gmm_no_pca	stress_skew	0.549	0.475	0.470	0.612
gmm_pca_95	stress_cv	0.737	0.843	0.978	0.099
gmm_pca_95	stress_iqr	0.732	0.841	0.982	0.074
gmm_pca_95	stress_mean	0.643	0.629	0.785	0.206
gmm_pca_95	stress_median	0.646	0.634	0.784	0.205
gmm_pca_95	stress_sd	0.781	0.873	0.983	0.100

gmm_pca_95	stress_skew	0.538	0.041	0.022	0.957
kmeans_no_pca	stress_cv	0.538	0.593	0.468	0.707
kmeans_no_pca	stress_iqr	0.599	0.690	0.624	0.546
kmeans_no_pca	stress_mean	0.901	0.916	0.928	0.923
kmeans_no_pca	stress_median	0.900	0.916	0.920	0.930
kmeans_no_pca	stress_sd	0.598	0.701	0.619	0.553
kmeans_no_pca	stress_skew	0.540	0.470	0.459	0.606
kmeans_pca_95	stress_cv	0.538	0.594	0.468	0.707
kmeans_pca_95	stress_iqr	0.599	0.690	0.624	0.546
kmeans_pca_95	stress_mean	0.901	0.916	0.928	0.924
kmeans_pca_95	stress_median	0.900	0.917	0.920	0.931
kmeans_pca_95	stress_sd	0.597	0.701	0.618	0.553
kmeans_pca_95	stress_skew	0.540	0.471	0.460	0.606

Table S4: D all surgeries clustering results pooled together

Mode	Label	Acc	F1	Sens	Spec
gmm_no_pca	stress_cv	0.702	0.477	0.502	0.778
gmm_no_pca	stress_iqr	0.633	0.155	0.246	0.694
gmm_no_pca	stress_mean	0.941	0.957	0.974	0.890
gmm_no_pca	stress_median	0.947	0.962	0.970	0.911
gmm_no_pca	stress_sd	0.503	0.370	0.442	0.500
gmm_no_pca	stress_skew	0.531	0.452	0.348	0.762
gmm_pca_95	stress_cv	0.611	0.302	0.428	0.682
gmm_pca_95	stress_iqr	0.725	0.237	0.287	0.790
gmm_pca_95	stress_mean	0.630	0.743	0.812	0.281
gmm_pca_95	stress_median	0.636	0.749	0.809	0.281
gmm_pca_95	stress_sd	0.633	0.380	0.423	0.710
gmm_pca_95	stress_skew	0.584	0.639	0.702	0.402
kmeans_no_pca	stress_cv	0.712	0.497	0.525	0.782
kmeans_no_pca	stress_iqr	0.630	0.154	0.246	0.690
kmeans_no_pca	stress_mean	0.944	0.959	0.974	0.898
kmeans_no_pca	stress_median	0.950	0.964	0.970	0.920
kmeans_no_pca	stress_sd	0.506	0.379	0.451	0.500
kmeans_no_pca	stress_skew	0.534	0.457	0.354	0.762
kmeans_pca_95	stress_cv	0.712	0.497	0.525	0.782
kmeans_pca_95	stress_iqr	0.630	0.154	0.246	0.690
kmeans_pca_95	stress_mean	0.944	0.959	0.974	0.898
kmeans_pca_95	stress_median	0.950	0.964	0.970	0.920
kmeans_pca_95	stress_sd	0.506	0.379	0.451	0.500
kmeans_pca_95	stress_skew	0.534	0.457	0.354	0.762

Table S5: E all surgeries clustering results pooled together

Mode	Label	Acc	F1	Sens	Spec
gmm_no_pca	stress_cv	0.616	0.696	0.570	0.772
gmm_no_pca	stress_iqr	0.535	0.628	0.529	0.551
gmm_no_pca	stress_mean	0.927	0.924	0.993	0.875

gmm_no_pca	stress_median	0.892	0.883	0.993	0.824
gmm_no_pca	stress_sd	0.520	0.619	0.519	0.523
gmm_no_pca	stress_skew	0.544	0.555	0.533	0.556
gmm_pca_95	stress_cv	0.788	0.864	0.872	0.506
gmm_pca_95	stress_iqr	0.799	0.869	0.894	0.528
gmm_pca_95	stress_mean	0.506	0.597	0.829	0.250
gmm_pca_95	stress_median	0.500	0.196	0.150	0.740
gmm_pca_95	stress_sd	0.837	0.894	0.915	0.605
gmm_pca_95	stress_skew	0.616	0.488	0.342	0.931
kmeans_no_pca	stress_cv	0.613	0.693	0.566	0.772
kmeans_no_pca	stress_iqr	0.538	0.631	0.533	0.551
kmeans_no_pca	stress_mean	0.930	0.927	1.000	0.875
kmeans_no_pca	stress_median	0.895	0.886	1.000	0.824
kmeans_no_pca	stress_sd	0.523	0.622	0.523	0.523
kmeans_no_pca	stress_skew	0.541	0.551	0.527	0.556
kmeans_pca_95	stress_cv	0.613	0.693	0.566	0.772
kmeans_pca_95	stress_iqr	0.538	0.631	0.533	0.551
kmeans_pca_95	stress_mean	0.930	0.927	1.000	0.875
kmeans_pca_95	stress_median	0.895	0.886	1.000	0.824
kmeans_pca_95	stress_sd	0.523	0.622	0.523	0.523
kmeans_pca_95	stress_skew	0.541	0.551	0.527	0.556

Table S6: F all surgeries clustering results pooled together

AB/G

Mode	Label	Acc	F1	Sens	Spec
gmm_no_pca	stress_cv	0.701	0.771	0.734	0.634
gmm_no_pca	stress_iqr	0.574	0.712	0.611	0.313
gmm_no_pca	stress_mean	0.616	0.729	0.609	0.491
gmm_no_pca	stress_median	0.621	0.732	0.612	0.491
gmm_no_pca	stress_sd	0.580	0.700	0.589	0.508
gmm_no_pca	stress_skew	0.528	0.534	0.684	0.422
gmm_pca_95	stress_cv	0.701	0.784	0.800	0.480
gmm_pca_95	stress_iqr	0.759	0.841	0.769	0.693
gmm_pca_95	stress_mean	0.677	0.786	0.722	0.361
gmm_pca_95	stress_median	0.672	0.782	0.719	0.342
gmm_pca_95	stress_sd	0.768	0.845	0.779	0.710
gmm_pca_95	stress_skew	0.545	0.326	0.293	0.715
kmeans_no_pca	stress_cv	0.630	0.698	0.615	0.657
kmeans_no_pca	stress_iqr	0.555	0.687	0.559	0.538
kmeans_no_pca	stress_mean	0.611	0.704	0.543	1.000
kmeans_no_pca	stress_median	0.613	0.705	0.545	1.000
kmeans_no_pca	stress_sd	0.541	0.674	0.552	0.492
kmeans_no_pca	stress_skew	0.524	0.491	0.573	0.491
kmeans_pca_95	stress_cv	0.630	0.698	0.615	0.657
kmeans_pca_95	stress_iqr	0.555	0.687	0.559	0.538
kmeans_pca_95	stress_mean	0.611	0.704	0.543	1.000
kmeans_pca_95	stress_median	0.613	0.705	0.545	1.000

kmeans_pca_95	stress_sd	0.541	0.674	0.552	0.492
kmeans_pca_95	stress_skew	0.524	0.491	0.573	0.491

Table S7: G all surgeries clustering results pooled together

Surgeon	Stress Samples	Non-Stress Samples	Total Samples	Stress (%)	Non-Stress (%)
G	527	88	615	85.7	14.3
C	859	445	1304	65.9	34.1
D	824	490	1314	62.7	37.3
E	219	103	322	68.0	32.0
A	1279	692	1971	64.9	35.1
B	1300	336	1636	79.5	20.5
F	152	192	344	44.2	55.8
Total	5160	2346	7506	68.7	31.3

Table S8: Class distribution for each surgeon used in the subject-wise LOSO

Evaluation Strategy	Stress Samples	Non-Stress Samples	Total Samples	Stress (%)	Non-Stress (%)
Subject-wise LOSO	5160	2346	7506	68.7	31.3
Random 70/30 Split	1549	704	2253	68.8	31.2

Table S9. Class distribution across evaluation strategies

Mode	model	seq_len	acc	f1	auc	sens	spec	Best model
Mean	CNN	1	0.87	0.91	0.95	0.90	0.82	CNN
Mean	LSTM	1	0.86	0.90	0.95	0.93	0.70	
Mean	CNN	2	0.89	0.92	0.95	0.86	0.96	CNN
Mean	LSTM	2	0.86	0.90	0.94	0.92	0.73	
Mean	CNN	5	0.87	0.89	0.94	0.82	0.96	Same
Mean	LSTM	5	0.87	0.90	0.94	0.85	0.91	
Mean	CNN	10	0.88	0.91	0.95	0.86	0.94	Same
Mean	LSTM	10	0.88	0.91	0.95	0.86	0.93	

Mean	CNN	20	0.86	0.89	0.94	0.84	0.89	LSTM
Mean	LSTM	20	0.88	0.91	0.96	0.84	0.96	

Table S10: Result with different sequence length and different models for 70\30

Mode	model	seq_len	acc	f1	auc	sens	spec	Best model
Mean	CNN	1	0.95	0.96	1.00	1.00	0.86	LSTM
Mean	LSTM	1	0.99	0.99	1.00	0.98	1.00	
Mean	CNN	2	0.97	0.98	1.00	1.00	0.93	Almost same
Mean	LSTM	2	0.97	0.98	1.00	1.00	0.91	
Mean	CNN	5	0.96	0.97	1.00	1.00	0.88	LSTM
Mean	LSTM	5	0.97	0.98	1.00	1.00	0.92	
Mean	CNN	10	0.95	0.96	1.00	1.00	0.86	LSTM
Mean	LSTM	10	0.98	0.99	1.00	1.00	0.95	
Mean	CNN	20	0.91	0.94	0.98	0.99	0.75	Almost same
Mean	LSTM	20	0.91	0.94	1.00	1.00	0.72	

Table S11: Result with different sequence length and different models for LOSO/A

Mode	model	seq_len	acc	f1	auc	sens	spec	Best model
Mean	CNN	1	0.87	0.91	1.00	0.83	1.00	LSTM
Mean	LSTM	1	0.90	0.94	1.00	1.00	0.50	
Mean	CNN	2	0.86	0.90	0.99	0.83	1.00	Almost same

Mean	LSTM	2	0.86	0.90	0.99	0.82	1.00	
Mean	CNN	5	0.87	0.91	0.98	0.84	1.00	LSTM
Mean	LSTM	5	0.89	0.93	0.99	0.86	1.00	
Mean	CNN	10	0.87	0.91	0.99	0.84	1.00	LSTM
Mean	LSTM	10	0.87	0.92	0.98	0.84	1.00	
Mean	CNN	20	0.85	0.90	0.97	0.83	0.96	LSTM
Mean	LSTM	20	0.85	0.90	0.98	0.82	1.00	

Table S12: Result with different sequence length and different models for LOSO/B

Mode	model	seq_len	acc	f1	auc	sens	spec	Best model
Mean	CNN	1	0.84	0.86	1.00	0.75	1.00	LSTM
Mean	LSTM	1	0.89	0.92	1.00	1.00	0.67	
Mean	CNN	2	0.92	0.93	1.00	0.87	1.00	LSTM
Mean	LSTM	2	0.94	0.95	1.00	0.91	1.00	
Mean	CNN	5	0.90	0.92	0.99	0.85	1.00	LSTM
Mean	LSTM	5	0.91	0.93	1.00	0.86	1.00	
Mean	CNN	10	0.93	0.94	0.99	0.89	0.99	CNN
Mean	LSTM	10	0.91	0.93	0.99	0.87	1.00	
Mean	CNN	20	0.89	0.91	0.98	0.84	0.98	LSTM
Mean	LSTM	20	0.91	0.93	0.99	0.87	0.98	

Table S13: Result with different sequence length and different models for LOSO/C

Mode	model	seq_len	acc	f1	auc	sens	spec	Best model
Mean	CNN	1	0.89	0.92	1.00	1.00	0.70	CNN
Mean	LSTM	1	0.89	0.91	1.00	0.83	1.00	
Mean	CNN	2	0.89	0.92	1.00	1.00	0.69	LSTM
Mean	LSTM	2	0.91	0.93	1.00	1.00	0.75	
Mean	CNN	5	0.91	0.93	1.00	1.00	0.75	Same
Mean	LSTM	5	0.90	0.93	1.00	1.00	0.73	
Mean	CNN	10	0.87	0.91	0.99	1.00	0.65	LSTM
Mean	LSTM	10	0.89	0.92	1.00	1.00	0.68	
Mean	CNN	20	0.87	0.91	0.96	0.99	0.61	LSTM
Mean	LSTM	20	0.88	0.92	1.00	1.00	0.64	

Table S14: Result with different sequence length and different models for LOSO/D

Mode	model	seq_len	acc	f1	auc	sens	spec	Best model
Mean	CNN	1	0.63	0.63	1.00	0.46	1.00	LSTM
Mean	LSTM	1	0.67	0.68	1.00	0.51	1.00	
Mean	CNN	2	0.66	0.67	1.00	0.50	1.00	Almost same
Mean	LSTM	2	0.65	0.66	1.00	0.49	1.00	
Mean	CNN	5	0.66	0.67	0.95	0.51	1.00	Almost same
Mean	LSTM	5	0.64	0.65	0.97	0.48	1.00	

Mean	CNN	10	0.68	0.69	0.94	0.53	1.00	CNN
Mean	LSTM	10	0.66	0.67	0.96	0.51	1.00	
Mean	CNN	20	0.69	0.70	0.96	0.54	1.00	CNN
Mean	LSTM	20	0.64	0.63	0.94	0.46	1.00	

Table S15: Result with different sequence length and different models for LOSO/E

Mode	model	seq_len	acc	f1	auc	sens	spec	Best model
Mean	CNN	1	0.80	0.71	1.00	0.55	1.00	CNN
Mean	LSTM	1	0.56	0.03	1.00	0.01	1.00	
Mean	CNN	2	0.83	0.77	1.00	0.63	1.00	Almost same
Mean	LSTM	2	0.85	0.80	1.00	0.66	1.00	
Mean	CNN	5	0.85	0.80	0.98	0.67	1.00	Same
Mean	LSTM	5	0.85	0.79	0.99	0.65	1.00	
Mean	CNN	10	0.87	0.83	0.99	0.71	1.00	CNN
Mean	LSTM	10	0.85	0.80	0.98	0.67	1.00	
Mean	CNN	20	0.90	0.87	0.98	0.78	0.99	CNN
Mean	LSTM	20	0.87	0.82	0.97	0.70	1.00	

Table S16: Result with different sequence length and different models for LOSO/F

Mode	model	seq_len	acc	f1	auc	sens	spec	Best model
Mean	CNN	1	0.40	0.47	1.00	0.31	1.00	LSTM

Mean	LSTM	1	0.64	0.73	1.00	0.58	1.00	
Mean	CNN	2	0.47	0.55	1.00	0.38	1.00	Almost same
Mean	LSTM	2	0.49	0.58	1.00	0.41	1.00	
Mean	CNN	5	0.47	0.55	1.00	0.38	1.00	Same
Mean	LSTM	5	0.46	0.54	1.00	0.37	1.00	
Mean	CNN	10	0.49	0.58	1.00	0.41	1.00	CNN
Mean	LSTM	10	0.46	0.54	1.00	0.37	1.00	
Mean	CNN	20	0.47	0.56	0.98	0.39	1.00	CNN
Mean	LSTM	20	0.44	0.52	1.00	0.36	1.00	

Table S17: Result with different sequence length and different models for LOSO/G
